# On the ocular findings in ochronosis: a systematic review of literature

**DOI:** 10.1186/1471-2415-14-12

**Published:** 2014-01-30

**Authors:** Moritz Lindner, Thomas Bertelmann

**Affiliations:** 1 Department of Ophthalmology, University of Bonn, Ernst-Abbe-Street 2, Bonn 53127, Germany; 2 Department of Ophthalmology, Philipps University Marburg, Baldinger-Street, Marburg 35043, Germany

**Keywords:** Hereditary ochronosis, Endogenous ochronosis, Alkaptonuria, Oil-drops, Eye, Opththalmology, Homogentisic acid, Ocular, Homogentisic acid oxidase deficiency

## Abstract

**Background:**

Ochronosis/Alkaptonuria is a tyrosine metabolism disorder where accumulation of homogentisic acid, in eye, skin, cartilage and several other connective tissues leads to a black pigmentation of the affected tissues. It is autosomal-recessive inherited in men with a frequency of 1-9/1,000,000. While it is clear that pigment deposits lead to joint destruction, renal stone formation and cardiac valvulopathy respectively, the significance of ocular findings is still unclear. We therefore aim to evaluate the frequency and clinical significance of ocular findings in ochronosis and discuss possible therapeutic options.

**Methods:**

Systematic review of literature via Medline and Web of Science. Only case reports in English, German, French, Spanish or Italian documenting detailed ophthalmologic examination were included.

**Results:**

Our search revealed 36 case reports including 40 patients. Average age at the onset of ocular signs was 40.6 years. The most frequent sign was symmetric brown sclera pigmentation present in 82.5 percent of the patients. “Oil-drops”, brown pigment spots in the limbus are generally considered pathognomonic but were a little less frequent (75 percent). Vermiform pigment deposits at the level of the conjunctiva or increased conjunctival vessel diameter is also frequent. We found an increased incidence of central vein occlusion and elevated intraocular pressure going along with chamber angle hyperpigmentation. Another condition observed twice is rapid progressive astigmatism attributable to corneoscleral pigment accumulation.

**Conclusion:**

Our observations suggest that ocular findings are of double relevance. First, characteristic ocular findings can anticipate the time of diagnosis and second, ocular findings may complicate to various conditions putting sight at risk. Opthalmologists and general physicians should be aware of both. Therapeutic options include protein restriction, administration of high dose vitamin C or nitisonone. Evidence for all of them is limited.

## Background

**Figure 1:**
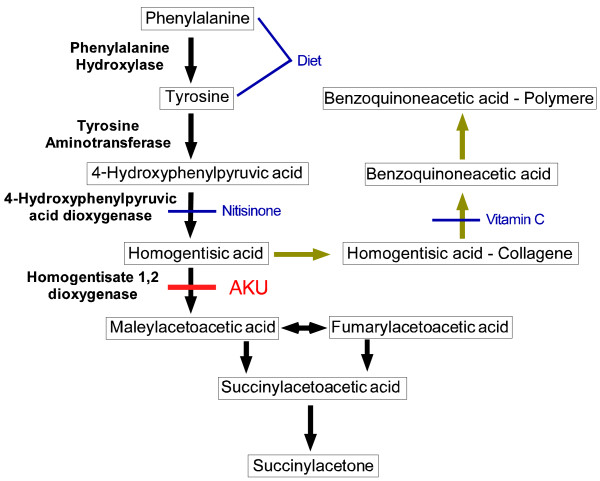
**Tyrosine metabolism pathway in health (black) and AKU (ochre) **[10]**,**[11]**.** Metabolites are drawn in Boxes, enzymes are given in bold. Red: Defect in AKU. Blue: Sites of possible therapeutic interventions.

Alkaptonuria (AKU/hereditary ochronosis/homogentisic acid oxidase deficiency/ORPHA56) is a rare autosomal recessive condition where tyrosine metabolism is disturbed [1,2]. Its frequency is estimated with 1-9/1,000,000 in overall population but it may reach much higher values in certain ethnic groups [3-5]. In AKU, homogentisic acid 1,2 dioxygenase (HGO, EC 1.13.11.5) is mutated [1]. Until today, 115 mutations are identified [6]. HGO converts homogentisic acid (2,5-Dihydroxyphenylacetic acid, HGA) – the third intermediate of tyrosine catabolism – to maleylacetoacetic acid (Figure 1, black arrows) [7,8]. In AKU, enzyme deficiency leads to a complete block within the tyrosine metabolism cascade [1]. As a consequence, HGA levels in plasma increase by a manifold [3]. A portion of the HGA is excreted in urine. When exposed to air, HGA oxidizes giving the urine a deep brown color [9,10]. Another portion of HGA accumulates in connective tissue over the years where it associates with collagen fibers, followed by non-catalytic polymerization and oxidation to benzoquinoneacetic acid (BQA, Figure 1, ochre arrows). Binding thus becomes irreversible resulting in melanin-like pigment deposits, macroscopic brown turning and dysfunction of the tissue [10-12].

While the genetic defect, resulting in brown discoloration of urine is referred to as alkaptonuria, the term hereditary ochronosis is applied for the tissue manifestation of the disease, classically including cartilage, skin and sclera [10,13]. Cartilage involvement is probably the most striking feature, accurately described by Rudolf Virchow who also developed the term “ochronosis” [14].

Clinically and radiologically ochronotic cartilage destruction closely imitates anklyosating spondylitis additionally affecting the big peripheral joints and making joint replacement necessary at young age [3,10]. Pigment accumulation further leads to ocular affection, skin darkening, cardiac valve impairment and several other less frequent affections [3,10,15]. Many clinical aspects of ochronosis have been assessed in detail [3] and a recent review aims to estimates their frequencies [15]. However, attempts to assess the nature of the ocular signs in ochronosis date back at least to the 1950s and are rather unsystematic [16-18]. Consequently also good estimates regarding the frequency of ocular findings are rare. Several authors claim that they are present in more than 2/3 of the cases reported in literature, suggesting that they could be of significant diagnostic value [16-20]. Recent case reports additionally propose that they might also be associated with pathologic conditions like glaucoma, progressive astigmatism and anterior uveitis [21-24].

The core of this work is formed by a systematic review of literature concerning the nature, frequency and clinical consequences of ochronotic changes in the eye. We will further discuss diagnostic, therapeutic and prognostic aspects from an ophthalmologic point of view.

## Methods

A Medline and Web of Science search was conducted using the term “(Eye AND alkaptonuria) OR (eye AND ochronosis) OR (ocular AND alkaptonuria) OR (ocular AND ochronosis) OR (ophthalmology AND alkaptonuria) OR (ophthalmology AND ochronosis)” on 12/Feb/2013. In our analysis only full-text available case reports or case series published in English, German, Spanish, French or Italian were included. Double reported cases were excluded.

To accurately estimate the frequency of the single ocular signs, articles not documenting a proper ophthalmologic examination were excluded from our analysis. Within the articles identified as relevant we checked for references that did not appear in our search results and included those in our analysis.

## Results

Our literature search revealed 74 articles. Within them we found 36 relevant case reports including 40 patients published between 1942 and 2012. Excluded articles and respective reasons are given in Table 1. The mean age of our patients was 61.0 (Standard deviation: ±9.5) years, with the youngest patient being 14 and the oldest 83 years old. The age at which the ocular findings first appeared was only given in eight articles [25-32] and was 40.6 (Standard deviation: ±9.8, range 25 to 54) years.

**Table 1:** Overview: articles found in medline/web of science search that were excluded from our analysis

**References**	**Reason for exclusion**	**n**
[33,34]	Full text not accessible	2
[35-39]	Not case report or case series	5
[40-47]	Not published in English, German, French, Spanish or Italian	8
[48]	Case double published	1
[49-51]	Not relevant	3
[12,52-69]	No documentation of proper ophthalmologic examination	20
Sum		39

### Clinical findings

**Figure 2:**
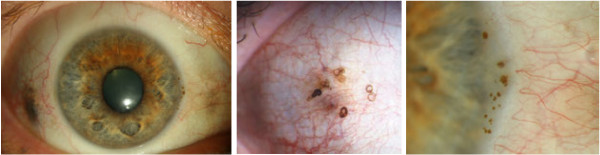
**Biomicroscopic findings in ochronotic eyes.** Left: Symmetric brown scleral pigmentation at 3 and 9 o’clock (w. permission [31]). Middle: “Vermiform” pigment accumulation in the conjunctiva (Courtesy of Dr. U. Hackethal [88]). Right: “Oil drops” of the corneal limbus (w. permission [31]). All images show the right eye.

The most common finding was hyperpigmentation of the sclera which was present in 33 patients [17,18,21-32,70-86]. This scleral pigmentation was mostly observed symmetrically to both sides of the cornea within the palpebral fissure having blurred margins (Figure 2, left, with permission [31]). In most reports it remains unspecified at which level of the sclera the pigmentations were seen. D’Alessandro et al. see them in the deeper layers of the sclera [70]. Conjunctival changes were also frequent (22 cases) but more heterogeneous in appearance [18,21-23,25-27,75-82,86-90]. There are four main types of appearance which may also occur in combination: 1) Small “vermiform” [88] or “tube-like” [76] pigment deposits (Figure 2, middle, with permission [88]) [18,27,76,81,86,88], 2) brown pingecula-like [81,87,89], 3) dot like [72,77] or 4) laminar structures [22]. Dilatated conjunctival vessels can be present and seemingly supply the pigmented areas [25,70,76,85,87,88]. Findings on the ocular surface are usually symptomless. “Sicca”-symptoms or foreign body sensation have only been reported twice in literature [31,89].

Pigment may also accumulate in the opaque portion of the limbus where it forms “oil drop”-like brown spots (Figure 2, right, with permission [31]), seen in 30 cases [17,18,21-23,25-29,31,70-81,86,88],[90,91]. In analog to the pigmentations of sclera and conjunctiva, also the limbal pigment drops follow a horizontal orientation. They are located at the level of the Bowman’s Membrane or slightly inferior to it [17,27,76,77]. In one case they were explicitly noted as the first feature of ochronosis in a 25 years old patient [27].

Severe progressive astigmatism has been reported twice [21,22]. It was attributed by the authors to ochronotic accretions that were integrated from the sclera into the limbus and may have lead to corneoscleral thinning. Both patients were in their 70ies when astigmatism began to develop and in both cases the axis of the cylinder was around 0° thus oriented in a right angle to the pigment. Astigmatism progressed by 6 dpt within two years in one case and by 7 dpt within 11 years in the other [21,22]. Ehongo et al. attempted a corneal transplant and bilateral thread placement. Both attempts did not bring enduring success, however re-progression was much slower after thread placement [21].

Hyperpigmentation of the chamber angles has been reported four times [23,31,70,76]. Several other findings have only been observed sporadically. They include brown pigmentation of the optic disc [91], the retina [28] and the vitreous [91]. In addition, pigment inclusions may occur at the iris [28,32,76] or fit to the posterior surface of the lens [31,90]. Table 2 summarizes the most common ophthalmologic findings.

**Table 2:** Frequency of the common signs present in the 37 patients summarized in this article

	**n**	**Percentage**	**References**
**Scleral pigmentation**	33	82.5%	[17,18,21-32,70-86]
**Oil drops**	30	75.0%	[17,18,21-23,25-29,31,70-81,86,88],[90,91]
**Conjunctival pigmentation**	22	60.0%	[18,21-23,25-27,75-82,86-90]
**Conjunctival vessels with increased diameter**	6	15.0%	[25,70,76,85,87,88]
**Pigmented chamber angle**	4	10.0%	[23,31,70,76]

Pigment in the chamber angles might lead to increased intraocular pressure (IOP) and thereby to glaucomatous lesions. In total, 15 articles commented on the IOP, eight reporting values above 21 mmHg [21,23,70,73,74,76,85,86]. Interestingly, in three of them the patients suffered from acute secondary glaucoma, always attributed to central vein occlusion [70,73,74]. Among the remaining cases, a primary glaucoma was only described in one case [23] while an increased intraocular pressure was documented, but not further discussed in two [76,85]. Noteworthy, gonioscopy is only described in two of the cases indicating elevated IOP. It revealed a hyperpigmented chamber angle each time [23,76].

There are two reports of acute recurrent anterior uveitis and ochronosis [18,24]. In the first one uveitis is claimed to be the initial manifestation of ochronosis [24]. In the second one the patient has had uveitis in history and the authors see no association with ochronosis [18]. Both patients suffered from unilateral uveitis. While it was described as side-alternating and non-granulomatous in first case [24], in the other case recurrent episodes of specific uveitis on always the same – now blind – eye were reported [18].

### Histopathology

**Figure 3:**
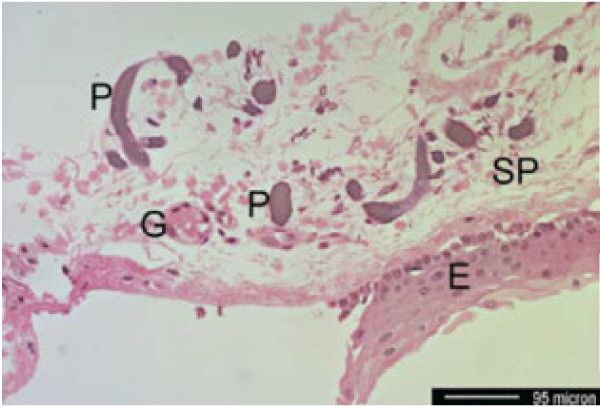
**Histopahtologic slice through a specimen obtained from the conjunctiva (Hematoxilin-Eosin staining).** P: Ochronotic pigment, E: Conjunctival epithelium, SP: Substantia propria, G: conjunctival vessel. Courtesy of Dr. U. Hackethal [88].

We found six detailed histopathologic descriptions of the whole bulbus [28,29,73-75,81] and four descriptions of ocular biopsies [27,82,88,89]. Ochronotic pigment is usually referred to as melanin-like which is true for the non-tinged tissue where it has a brown to olive, non-birefringent appearance. Histochemically, it rather behaves like elastin as being positively stained by van Gieson or Movat-pentachrome but not by Fontana [29,73,75,81,89]. In hematoxilin-eosin it is seen as light wine-red to eosinophilic but still with a pale olive appearance [81,88] (Figure 3, with permission [88]).

In accordance to the clinical findings, also histologically the central cornea is usually clear. Though phagocytised pigment may well occur inside central endothelial cells [29]. The limbal “oil-drops” are correlated by globular accretions adjoining the Bowman’s membrane or infiltrating the anterior stroma [29,73,75,81]. Kampik et al. describe them as rather small granules that are located around or even inside the keratocytes [73].

All parts of the sclera can be affected by ochronotic pigment granules [73-75]. However, the major part of the ochronotic pigment is found to reach from the insertion of the rectus muscles towards pars-plana [29,73-75,81]. Pigment can be either extracellular, forming beads along collagene fibers, or intracellular in macrophages and fibrocytes [28,75,81]. Areas of strongest pigment accumulation were associated with degeneration of sclera fibers [28,29,75]. Inflammatory reactions were not seen in any of the histopathologic works assessed here. Their absence is explicitly mentioned in two works [28,82].

The structures of chamber water production and drainage are usually not commented on. However, Allen et al. describe deposit of pigment dust in the ciliar process and ciliar body [75]. The only statements on chamber angles are made by Stürmer et al. and Rones et al. who describe them as open but do not state if pigmented or not [28,29].

### Initial diagnosis

In the cases analyzed here, the initial diagnosis was often not determined correctly. In four cases, the initial diagnosis was melanoma [30,72,76,79]. In another case, a patient was diagnosed as anklyosating spondylitis and referred to the ophthalmologist to exclude an accompanying iridocylitis [79]. Finally, in a case where conjunctival “vermiform” findings were the leading sign, parasitosis had been suspected [88].

## Discussion

AKU is an extremely rare metabolic disorder caused by autosomal recessive inherited mutations in the HGO-gene, an enzyme of tyrosine catabolism [1,2]. The defect of this enzyme results in accumulation of HGA in tissue and urine. In tissue HGA is oxidized to BQA which giving it a dark brown colour [1,3,10]. It is well established that this goes along with tissue dysfunction, resulting in arthropathy, calcification of cardiac valves and renal stone formation [3,10]. In contrast, ocular signs are poorly investigated. To our knowledge, this is the first systematic review of the ocular manifestations of ochronosis.

### Clinical findings and complications

Ocular signs are present in at least 2/3 of the patients affected by hereditary ochronosis [16-20]. In our review scleral hyperpigmentations were the most common finding (Figure 2, left). Slightly less frequent were oil-drop like pigment accumulation in the opaque limbus and pigment accumulation in the conjunctiva (Figure 2, middle and right). These results are in agreement with estimations made earlier by other authors [10,16,19]. It must be noted that scleral findings are easily visible even without the help of biomicroscopy and possibly patients having noticed changes on their eyes are more likely to present to an ophthalmologist than those who have not. The real frequency of corneal and other clandestine findings may therefore be underestimated.

Due to their characteristic appearance, corneal “oil-drops” are of high diagnostic significance. In agreement with others [10,18], we think that “oil-drops” are pathognomonic to ochronosis.

The scleral pigmentations are histologically shown to affect the whole depth of the sclera between rectus muscle and pars plana. Here an association with scleral fibres was observed, resulting in degenerative changes. Clinically pigmentation was observed to overcome pars plana and continue to the limbus. Two publications report a rapid progressive astigmatism that developed at an unusual old age. Pigment continuing into the limbus was present and the axis of astigmatism was shifted by 90 ° towards the pigment orientation [21,22]. In synopsis, these clinical and histological reports suggest that ochronotic pigment may affect the stability of the sclera and corneal margins likewise, leading to a cylinder-like deformation of the cornea.

Our analysis further revealed that pigment has also been seen in the chamber angles [23,31,70,76]. It is usually referred to as being only of moderate intensity. However, it seems possible that such pigment could lead to an IOP increase. With IOP being elevated in 8 out of 14 articles reporting on it, also the frequency seems clearly elevated. Obviously, this observation might be biased by the fact that IOP is more likely to be reported in a case report if being pathologic. But even suggesting that IOP was normal in all other cases, a frequency of 8/40 would still be very high.

We were surprised to also find three cases of central vein occlusion among the case reports [70,73,74]. Authors of all three cases did not correlate central vein occlusion with ochronosis, it was rather seen as coincidence, possibly because none of them seemed to be aware of the case description by the others. Although a pathogenetic link is hard to see, it is difficult to believe that a frequency as high as three out of 40 cases should really be coincidence.

Two reports document acute recurrent unilateral anterior uveitis together with ochronosis [18,24]. It was side alternating and non-granulomatous in the one, unilateral and granulomatous in the other case, suggesting distinct etiologies. As existing histologic evidence [28,82] and the knowledge about the pathogenesis [10] of ochronosis argue against any immunologic component, we consider it to be very unlikely that ochronosis could be associated with any condition of uveitis.

We have finally shown that ochronotic pigment can also accumulate at uncommon locations, such as the corneal endothelium, the posterior surface of the lens, the iris, the vitreous, and the optic disc.

### Diagnosis

Only a minority of patients is diagnosed due to the earliest sign of alkaptonuria: darkening of urine [3,10,15]. Most frequently diagnosis is set in adulthood when ochronosis becomes symptomatic [10,15]. What is the value of ocular findings for establishing the diagnosis? Early works suggest scleral pigmentation to be the earliest sign of ochronosis, presenting in the mid-twenties [10,18]. However, in the cases analysed here ocular pigmentation occurred at a mean age of 40.6 years. This seems old, however ochronosis is diagnosed in average at the age of 56 years, according to a recent review [15] and a recent case series [20] dealing with ochronosis in general. This suggests that awareness of ocular signs can anticipate the diagnosis by about 15 years.

Although ocular findings are very characteristic, a number of differential diagnoses should be thought of. Exogenous ochronosis (caused by local contact to a causative agent like resorcinol, hydroquinone, mercury, phenol, picric acid) has to be considered. It results in symptoms limited to the contact area, usually at the skin [92] but also exogenous ocular ochronosis may occur [49,93-95]. Anamnesis and ocular aspects are extremely different from hereditary ochronosis: The cornea is more frequently involved and also the central cornea is affected. Consequently vision is severely impaired [49]. Besides differential diagnoses include hyaline degenerations, Addison’s disease or scleral melanosis which can all cause scleral hyperpigmentations but do not affect the cornea. Choroid melanoma was the most popular initial diagnosis in the articles we analyzed. This differential diagnosis is of high relevance because it may not be overseen. But, vice versa, misdiagnosis of ochronosis as melanoma may also be fatal: It has lead to enucleation of a patient’s last eye [52]. Finally, pigmentation of the chamber angle could be due e.g. to a pigment-dispersion syndrome or ocular melanosis [16]. However, there is no evidence that oil-drop like plaques of the corneal limbus are present in any other condition but ochronosis.

Once the diagnosis is clinically set, it can be fortified by the detection of homogentisic acid in plasma or urine [1,15,96] and confirmed by genetic analysis. AKU mutations reported so far were summarized by Zatkova et al. [6].

In our literature review we found two striking examples underlining the relevance of the opthalmologist in the diagnostic process of this disease. In one instance an ochronotic eye was enucleated for suspecting melanoma, and diagnosis of ochronosis was set by the pathologist [52]. In another instance a patient was apparently long treated under the faulty diagnosis of anklyosating spondylitis. Correct diagnosis was not established until he was referred to an ophthalmologist to check for an eventual uveitis [79].

### Treatment

Causative therapy of ochronosis must aim to reduce the creation of harmful BQA. This purpose could be fulfilled by three different approaches (Figure 1, blue notes): First, nitisonone (2-(2-nitro-4-trifluoromethylbenzoyl)-cyclohexane-1,3-dione [NTBC]), inhibiting 4-hydroxyphenylpyruvate-dioxygenase could block tyrosine metabolism on an earlier step, reducing the generation of HGA [97]. Second, vitamin C could exhibit an antioxidant effect on HGA and decrease its non enzymatic conversion to BQA [98,99]. Third, restriction of tyrosine and phenylalanine could decrease their plasma levels and thereby the generation of HGA [10]. Noteworthy these treatment options have been shown to reduce HGA and BQA levels respectively. However an effect on clinical symptoms or disease progression has yet not been evidenced. Therapeutic attempts by the ophthalmologist should therefore mainly cover the treatment of ocular complications. Concerning correction of rapid progressive astigmatism, classical therapeutic options are condemned to fail, as the reason for its development is located in the corneoscleral margin, which remains unaffected in any type of corneal transplant or crosslinking. Contact lenses are usually not well tolerated in the advanced age where ochronotic astigmatism develops. The attempt of a corneal transplant has only been documented once and brought only a short amelioration before astigmatism started to advance again [21]. A subsequent bilateral thread-positioning brought also only transient relief, but progression afterwards was slower suggesting that thread-placement might be an appropriate therapeutic option. For ochronosis-induced glaucoma Bacchetti et al. showed that trabeculectomy can be a successful while topic multi-medication was inefficient [23].

### Prognosis

Prognosis quo ad vitam is good. Life expectancy is believed not to differ obviously from healthy people [10,100]. Ochronosis goes along with significant physical impairment and increased frequency of surgery at young age [3] and both are associated with decreased life expectancy. It is thus possible that ochronosis could in fact limit life expectancy via this detour which was overseen due to the low number of patients. Quo ad vistam, our work for the first time shows that ochronosis has several sight threatening complications, including astigmatism, central vein occlusion and glaucoma.

## Conclusion

In conclusion, ocular findings are frequent in patients suffering from ochronosis. The pathognomonic “oil-drop” lesions of the limbus can serve as a valuable diagnostic hint. As ocular signs occur early in the development of the disease, they may anticipate the time of diagnosis and thereby open a therapeutic window. Astigmatism, glaucoma and central vein occlusion are three conditions frequently seen in patients with ochronosis. As these put vision at risk, the general physician and the ophthalmologist should be aware of them. Therapeutic options include nitisinone and antioxidant administration. Their use should be considered and started in adolescence.

## Abbreviations

AKU: Alkaptonuria; HGO: Homogentisic acid 1,2 dioxygenase; HGA: Homogentisic acid; IOP: Intraocular pressure; BQA: Benzoquinoneacetic acid.

## Competing interests

ML: Commercial Relationships: Heidelberg Engineering, Germany; Carl Zeiss Meditec, Germany; Optos, UK. All Code F (Financial Support). TB: The author declares that he has no competing interests.

## Authors’ contribution

ML: Literature research, editing of the manuscript. TB: Literature research, senior supervision, correction of the manuscript. Both authors read and approved the final manuscript.

## Pre-publication history

The pre-publication history for this paper can be accessed here:

http://www.biomedcentral.com/1471-2415/14/12/prepub
